# The Potential of Films as Transmucosal Drug Delivery Systems

**DOI:** 10.3390/pharmaceutics15112583

**Published:** 2023-11-04

**Authors:** Ana Clara Wada de Carvalho, Natália Floriano Paiva, Isabella Kriunas Demonari, Maíra Peres Ferreira Duarte, Renê Oliveira do Couto, Osvaldo de Freitas, Fabiana Testa Moura de Carvalho Vicentini

**Affiliations:** 1 School of Pharmaceutical Sciences of Ribeirão Preto, University of São Paulo, Brazil. Av. Café, Ribeirão Preto 14048-900, SP, Brazil; ana.wada.carvalho@usp.br (A.C.W.d.C.);; 2 Campus Centro-Oeste Dona Lindu (CCO), Universidade Federal de São João del-Rei (UFSJ), Divinópolis 35501-296, MG, Brazil

**Keywords:** buccal drug administration, drug delivery systems, nasal administration, ocular administration, polymers, transmucosal administrations, vaginal administration

## Abstract

Pharmaceutical films are polymeric formulations used as a delivery platform for administration of small and macromolecular drugs for local or systemic action. They can be produced by using synthetic, semi-synthetic, or natural polymers through solvent casting, electrospinning, hot-melt extrusion, and 3D printing methods, and depending on the components and the manufacturing methods used, the films allow the modulation of drug release. Moreover, they have advantages that have drawn interest in the development and evaluation of film application on the buccal, nasal, vaginal, and ocular mucosa. This review aims to provide an overview of and critically discuss the use of films as transmucosal drug delivery systems. For this, aspects such as the composition of these formulations, the theories of mucoadhesion, and the methods of production were deeply considered, and an analysis of the main transmucosal pathways for which there are examples of developed films was conducted. All of this allowed us to point out the most relevant characteristics and opportunities that deserve to be taken into account in the use of films as transmucosal drug delivery systems.

## 1. Introduction

Films are thin sheets composed of one or more polymers that may or may not contain a plasticizer. They can be applied in the pharmaceutical field as drug delivery systems or as dressings applied directly to wounds to enhance healing [1]. Mucoadhesive films, especially buccal ones, are a well-accepted dosage form among patients and prescribers, especially for pediatric and geriatric patients. When compared to buccal tablets, they are more thin, flexible, and therefore more comfortable, in addition to allowing the formulation to be cut to the desired size. The films can also be formulated for either a local or systemic action, avoiding the first pass effect, and can be produced with several layers, allowing a controlled and targeted drug release in addition to the incorporation of components with physical–chemical incompatibilities [2,3].

This review provides an overview of the use of films as transmucosal drug delivery systems and critically discusses issues related to their composition, the theories of mucoadhesion, and the methods of production. An analysis of the main transmucosal pathways (buccal, nasal, ocular, and vaginal) as well as the characteristics of the mucosa and examples of films for the pathway are also shown.

Within this comprehensive and integrated approach, which was based on the discussion of recently published studies, we reinforce that films are versatile and attractive for transmucosal drug delivery and deserve to be continuously explored to improve their applicability.

## 2. Classification of Films

### 2.1. Polymers

The stage of selecting the appropriate reagent plays an important role when defining the therapeutic objectives of a film-forming formulation, since the polymer can interfere with characteristics such as drug incorporation efficiency, drug release kinetics, the mucus adhesion mechanism, and formulation stability. For example, the swelling behavior and diffusional characteristics of polymers across different pH ranges are critical points for drug release, mucoadhesiveness, and therapeutic performance. Important polymer parameters such as biocompatibility, long-term stability, and low immunogenicity, among others, must be considered when selecting an appropriate polymer [4].

**Figure 1:**
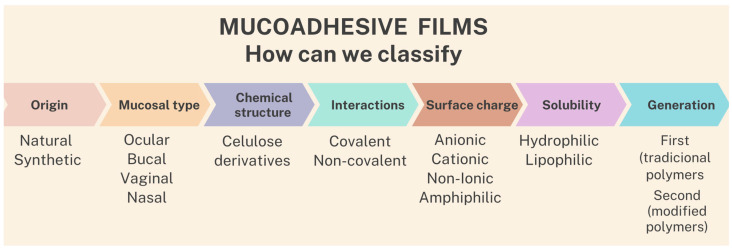
Classification of polymers used in the development of transmucosal films.

Polymers that are used for the production of mucoadhesive films can be classified according to: (1) origin—natural or synthetic; (2) type of applied mucosa—ocular, buccal, and vaginal, among others; (3) chemical structure—cellulose derivatives, among others; (4) interactions—covalent or non-covalent; (5) surface charge—anionic, cationic, non-ionic, and amphiphilic; (6) solubility—hydrophilic or lipophilic; and (7) generation—first or second generation [5,6], as shown in the scheme in Figure 1.

Polymers of the first generation are also called traditional non-specific polymers, which form non-covalent bonds between their functional groups and mucus substructures (mucins). In this context, these polymers present a non-specific adhesion to the mucus, with a consequent short retention time and rapid elimination due to the high volume of mucus. In general, the main advantage of the first-generation polymers is their simple production procedure that does not require many synthesis steps; however, they are characterized by the low mucoadhesion of the weak van der Waals binding forces and ionic interactions [7].

Among the first-generation polymers are the anionic, cationic, and non-ionic subsets. Regarding the non-ionic polymers, there are several hydroxypropyl methylcellulose (HPMC) types available that vary widely in terms of degree of substitution, molar substitution, nominal viscosity, and hydrophilicity [8,9]. In general, non-ionic polymers have a lower mucoadhesion strength than cationic and anionic ones, since their adhesion is due to the polymer interpenetration and there are no electrostatic interactions with mucin [5,6]. Nevertheless, low-viscosity HPMCs (e.g., HPMC K100 LV) have been yielding buccal films with suitable mucoadhesive properties [10,11,12].

Anionic polymers are the most widely used due to their higher mucoadhesion and lower toxicity. This class is determined by the presence of carboxyl and sulfate functional groups that, when in contact with a vehicle whose pH value is greater than the pKa of the polymer, result in a negative surface charge of the film [13]. According to Brannigan and Khutoryanskiy [14], regarding the adhesion mechanism of these materials, there is still disagreement in the literature about the effect and ideal pH ranges. Examples of anionic materials are those of natural origin, such as alginate, carboxymethylcellulose (CMC), and pectin, which have carboxylic acid as side groups. However, in recent years, there has been a predominance of the use of synthetic polymer poly (acrylic-acid)—PAA, which is synthesized via free radicals or via controlled polymerization of acrylic acid; the most common example being currently commercialized is Carbopol, which presents PAA-reticulated networks with allylpentaerythritol or allyl sucrose [14]. PAA and CMC have excellent mucoadhesive characteristics due to the formation of strong interactions and hydrogen bonding with mucin [15].

Cationic polymers also exhibit a strong ionic interaction with mucin, with chitosan as a widely used example [6]. This group of polymers present a strong mucoadhesion given by the ionic interactions between the polymer and the anionic structures of the mucus, such as the sialic acid groups [16,17]. Chitosan is a cationic polysaccharide produced by the deacetylation of chitin, which stands out for its biocompatibility, biodegradability, and favorable toxicological characteristics. While PAAs bind to mucus through hydrogen bonds, the chitosan forms ionic interactions between the primary amino functional groups and the sialic acid and sulfonic acid of the mucus (in addition to the hydrogen bonds that form between the hydroxyl and amino groups with the mucus). Furthermore, there is still the possibility of incorporating various chemical groups into the chitosan structure, particularly at the C-2 position, which allows the formation of new polymers with different functionalities [18]. As an alternative to chitosan, there are reports of studies on the development of aminated cellulose derivatives as well as the use of poly(L-lysine) (PLL), a polyamine derivative that also exhibits significant mucoadhesive properties [14,19]

However, due to the difficulties in the processing, formulation, and commercial availability of PLL, chitosan still predominates as the most used cationic polymer. In addition to natural polymers, there are also non-degradable synthetic ones, such as poly(allylamine) hydrochloride (PAH) and poly(2-dimethylamino)ethyl methacrylate (PDMAEMA). These are produced via radical polymerization techniques (for example, via free radical addition) and exhibit enhanced mucoadhesive properties in acidic environments due to the protonation of the amine derivatives. These materials can be easily incorporated into nanogel, liposome, and film formulations; however, in the case of PAH, one must consider the toxicity issues that restrict its application [14,19].

The second generation of polymers, called thiolates or thiomers, began to be developed at the end of the 1990s. These polymers are obtained by immobilizing sulfhydryl ligands in a polymeric skeleton through the formation of amide and amidine bonds. With this, several aspects of the formulation can be improved, such as the capacity for controlled drug release, increased permeation, and mucoadhesion. The increased process of adhesion to the mucus is due to the formation of a disulfide bond between the thiol group of the polymer and the cysteine-rich mucus [7].

Unlike the previously mentioned first-generation polymeric platforms, certain second-generation polymers are less susceptible to mucus clearance, so they bond directly to mucosal surfaces and are thus called “cytoadhesives”. While the first generation is characterized by non-covalent secondary interactions, the covalent binding mechanisms involved in the second-generation mucoadhesive platforms result in interactions that are less susceptible to changes in ionic strength or physiological pH [15].

Thiomers can be defined as polymers containing thiol functional groups, so they can be prepared by conjugating conventional mucoadhesive polymers with molecules that present thiol functionality. Examples include poly(acrylic acid)/cysteine, chitosan/N-acetylcysteine, alginate/cysteine, chitosan/thioglycolic acid, and chitosan/thioethylamidine [20]. In addition, lecitin has also been used as a cytoadhesive material that can bind to mucosal surfaces, as it belongs to a group of structurally diverse proteins and glycoproteins that can reversibly bind to specific carbohydrate residues. They are naturally occurring proteins that play a role in the process of biological recognition involving cells and proteins; for example, some bacteria use lecitins to attach to host organism cells during infection [21].

Currently, thiolated polymers are the most widely used second-generation mucoadhesives, but there are still other strategies under development that promote increased mucoadhesion and demonstrate some advantages over polymeric thiomers. Examples include polymers that allow the addition of catechol-, boronate-, acrylate-, methacrylate-, maleimide-, and N-hydroxy(sulfo)succinimide-ester groups. However, more research will be needed to determine the relative strength of adhesion of these polymers that are prepared with different strategies and whether they can be used for the development of new transmucosal drug delivery systems [14].

### 2.2. Plasticizers

In general, films are composed of polymers and plasticizing agents, the latter being necessary additives for adequate mechanical properties of a material regarding its manufacturing procedure and purposes. This property is obtained through the introduction of a small plasticizer molecule that is miscible with the film-forming polymer, allowing moderation of the polymer–polymer interaction through the interaction of the polymer with the plasticizer and thus promoting greater mobility of the polymeric chain. As a consequence, there is a reduction in the glass transition temperature and tensile strength, as well as an increase in the film elongation [22].

The formation of a polymeric film with desirable mechanical characteristics depends on the compatibility of the plasticizer with the polymers as well as an appropriate amount and number of binding groups; e.g., free hydroxyl groups [23]. As an example of the application of plasticizing agents, native alginate films need plasticizers such as polyols in their formulation, as they are very rigid. Thus, several polyol-plasticizers have already been studied and used in the production of formulations containing alginate film through the solvent casting method, among them glycerol, sorbitol, and xylitol. Glycerol is the most used plasticizer for alginate films [24].

Thus, plasticizers are essential components for the formulation of drug delivery systems from films, as it is possible to obtain films from them that present greater polymer flexibility by increasing the intermolecular separation of the polymer molecules after the loosening of resistance of the intermolecular forces, which also results in better patient compliance and adherence to pharmacotherapy. Studies report that in addition to acting as internal lubricants that reduce the intermolecular attraction of the polymer, they are also able to increase bioadhesiveness [25]. In this sense, Gal and Nussinovitch [26] reported that the presence of a plasticizer in transdermal patches exhibited additional bioadhesion to skin flexibility.

Suitable plasticizers for the film are selected according to their characteristics of biocompatibility; compatibility between the plasticizer, polymer, and drug; and drug release suitability. Thus, depending on the film purpose, formulation scientists have a broad range of hydrophilic, hydrophobic, and amphiphilic plasticizers from which to choose. In this context, polyethylene glycols (PEGs) and triethanolamine stand out for their high plasticizing effect; polyethylene glycols are highly biocompatible, but their miscibility decreases with an increase in the molecular weight. Studies indicate that PEG 400 is a more effective plasticizer when compared to PEG 1000 for HPMC films [25]. According to Sun et al. [27], glycerol, PEG-400, and sorbitol are frequently used as hydrophilic plasticizers in the formulation of films due to their good compatibility and solubility in water, which allows the films to overcome fragility and present desirable characteristics of elasticity and flexibility.

When developing multi-layered films, a backing layer formed by less polar polymers is often present. Herein, the hydrophobic plasticizers, in addition to yielding the desired mechanical properties, can preclude either the dissolution or bulk erosion of the release layer and contribute to ensuring a targeted and controlled drug release and permeation through the mucosa [28]. This is required for enhancing the outcomes of several medical procedures; e.g., buccal local anesthesia [11,12]

Likewise, hydrophobic plasticizers can be used in the formulation of monolithic films intended to provide a sustained drug release profile for a long time span, such as in vaginal and ocular administration. Acetate esters such as glyceryl triacetate (triacetin), triethyl citrate, and acetyl triethyl citrate; phthalate esters (e.g., diethyl phthalate and dibutylphthalate); and acetylated monoglycerides are widely used for these purposes [29,30,31,32].

Formulators also should consider that the drug exerts a plasticizing effect in the polymeric films by itself, which can be explained along the same lines as the aforementioned for any other small substance displaying binding functional groups. There is convincing evidence that increasing the drug loading correlates with a decrease in the glass transition temperature of polymers [33], which in turn correlates with a decrease in the tensile strength and tensile modulus of films [33,34]. Therefore, owing to this likely synergic effect, the amount of both the plasticizer and drug in the film should be set while seeking to balance the ideal mechanical properties and improved treatment outcomes.

Finally, it is worth mentioning that the study of the best polymers, plasticizers, and drug loading for the formulation is of paramount importance, since the same plasticizer may have different effects on different polymers [35]. For instance, mannitol can make HPMC films more resistant, while glycerol can make them more plastic [34]. Feasible strategies to be used in this pursuit are the Quality by Design (QbD) approaches such as the Design of Experiments (DoE) and Response Surface Methodology (RSM) [34,36].

## 3. Theories of Mucoadhesion Process

The walls of our body cavities, such as the gastrointestinal and respiratory tracts, are coated with moist surfaces called mucous membranes. These membranes present in their composition a layer of connective tissue (called the lamina propria), above which is located an epithelial layer characterized by the presence of a mucus layer. The epithelium has goblet cells that are able to secrete mucus directly onto epithelial surfaces as well as specialized glands (for example, the salivary glands) that also secrete mucus. Mucus can be present as a gel layer adherent to the mucosal surface or as a soluble or suspended luminal form; its main components are mucin glycoproteins, lipids, inorganic salts, and water. Mucins stand out in their importance due to their cohesion and adhesion properties. It is also worth mentioning that the mucus layer’s thickness is variable according to the different surfaces and that its main functions in the body are to protect and lubricate [37].

The term bioadhesion relates to the attachment of a drug delivery system to a particular site in the body, which may be an epithelial tissue or a mucosal coating on the surface of the tissue. When it comes to attachment to a mucus layer, the process can be referred to as mucoadhesion, characterized by the interaction between the mucin surface containing mucin and a polymeric material [38]. Thus, mucoadhesion stands out in drug administration, as it allows the retention of a formulation for local action as well as for systemic action. Mucoadhesive materials are thus used to deliver therapeutic agents themselves but also to coat and protect damaged tissues, such as gastric ulcers and oral mucosal lesions, and to act as lubricating agents in the oral cavity, eyes, and vagina [37].

The mucoadhesion mechanism is therefore a complex process in which the literature reports the involvement of two main phases: the contact phase and the consolidation phase. In the first one, the pharmaceutical form comes into contact with the mucosa, and this process is followed by wetting, so the material’s ability to hydrate and spread the interfacial forces between it and the mucus become essential factors in this process. The second stage is characterized by several physical–chemical interactions that occur between the formulation, the mucus, and the mucous membrane that allow prolonging of the adhesion, since in this stage the physical entanglement and the chemical bonds are the main factors that affect this adhesion process. In this context, there are some described theories that explain this mucoadhesion process that will be described below [39]. In general, the literature proposes five theories that should be considered as different phases of a single process that is characterized by multiple stages of interaction between the formulation and the mucus [15,20,40].

### 3.1. Absorption Theory

According to this theory, the adhesion process occurs through the formation of interfacial chemical bonds between the polymer and the mucus [40]. In this context, the resulting interactions can be of the primary bonding (covalent, metallic, and ionic bonds) or secondary bonding type (hydrogen bonds, hydrophobic interactions, and van der Waals forces), the latter being the most desirable and important for the process of mucoadhesion, as they are not permanent [15,41]. Secondary bonds of the van der Waals type are the most common, but despite being weaker, when present in larger amounts, they promote greater adhesion. In addition, there is also the formation of chemical bonds across the interface, a process called “chemisorption”, in which adhesion occurs through the formation of a strong covalent bond. In general, in this theory, adhesion is the result of primary and secondary interactions that form after initial contact between the polymer surface and the mucosa [42].

### 3.2. Electronic Theory

The electronic theory describes mucoadhesion as a process resulting from the transfer of electrons due to the different electrical properties of the mucus and the polymer [43]. This electron transfer process occurs through the contact of mucus glycoproteins with the mucoadhesive material, which leads to the formation of electrical double layers at this interface and consequently generates attractive electrostatic forces [39,41]. Thus, in this theory, it can be assumed that the strength of the mucoadhesion will depend on the forces of attraction within this electronic double layer (formed between the positively charged polymer and the negatively charged mucous membrane) as opposed to the high resistance of the secondary chemical interactions proposed in other theories [42,44].

### 3.3. Fracture Theory

It is known that the energy to break the adhesive bond is greater when the polymer has longer chains of polymeric networks and a lower degree of crosslinking [40,45]. Therefore, the application of this theory is appropriate to calculate the separation force of rigid or semi-rigid polymers from the adhered surface, in which the polymeric networks are not able to penetrate the mucus layer, since the theory does not consider interpenetration or diffusion of these chains [37,46]. As a result, through the calculations, it is assumed that the resistance to fracture will be equivalent to the adhesive force [38].

### 3.4. Wetting Theory

Based on the wetting theory, the penetration of mucoadhesive polymers occurs through the irregularities present on the surface of the mucosa, which is applicable to liquid and semi-solid systems. In this way, the adhesive capacity of the material depends on its wettability characteristic; that is, it is related to the degree of contact that a liquid might maintain with a solid surface as a result of intermolecular interactions [40,47]. This theory therefore applies to systems that have an affinity for the surface in such a way that they spread over it, and this adequate spreadability can be determined by means of measurement techniques such as the contact angle. In general, the greater the affinity and spreadability, the smaller the contact angle, with values equal to or close to zero being appropriate [46]. In this sense, the adhesion of the formulation to the mucosal surface is due to intermolecular interactions and the surface tension forces between the liquid and the mucosa, with spreadability being derived from the balance between the adhesive forces (liquid with the surface) and cohesive forces (liquid to liquid) [42].

### 3.5. Diffusion Theory

This theory describes the interpenetration of both polymer and mucin chains to a depth sufficient to form a semi-permanent adhesion. It is suggested that the adhesion strength increases with the degree of penetration of the polymeric chains, which depends on the diffusion coefficient, flexibility, and nature of these mucoadhesive chains, mobility, and contact time. Penetration of polymeric chains into the mucus network is driven by concentration gradients until equilibrium penetration depth is reached, at which point the mucus substrate and mucoadhesive chains move along their respective concentration gradients in opposite phases, with the depth of diffusion dependent on the diffusion coefficient of both phases. The diffusion coefficient in this context depends on the molecular weight and crosslinking density of the polymeric network. An interpenetration depth of 0.2 to 0.5 µm is necessary for an efficient mucoadhesion [37,38,46]. As a conclusion, a stronger mucoadhesive bond will be formed when there is a better mutual solubility between the polymer and the mucus [48].

## 4. Film Production Methods

There are several methods available for film production; however, the most used nowadays are solvent casting, electrospinning, hot-melt extrusion, inkjet printing, and 3D printing. In this section, a brief explanation of the operation of these methods will be given, presenting the limitations of each one and its advantages in relation to other production methods.

### 4.1. Solvent Casting

Because it is a simple and low-cost manufacturing process, solvent casting is the most used method for film preparation. The technique consists of preparing a homogeneous solution or suspension of the components, which must be deaerated in case of bubble formation, then an appropriate volume of the solution or suspension is transferred to a mold in which the formulation will be dried, leading to solvent evaporation and film formation [2,3,49]. The molds used for the drying process are made of aluminum, glass, Teflon, polyethylene, plastic, or silicone and can be individually sized to obtain films in the planned size and format [30] or in a larger size than intended. In the latter case, an additional cutting step is required to obtain the desired final dosage [3]. The solvent casting technique is versatile and can be used for both laboratory and industrial scales.

During the manufacture of films via the solvent casting method, it is important to control the rheological properties of the solution or suspension, to avoid trapped air bubbles, and to guarantee the uniformity of the content [3], since these variables interfere with the quality of the produced film. The viscosity of the solution or suspension to be melted influences the drying rate, the uniformity of the drug, and the physical appearance of the films so that the increase in viscosity interferes with the components’ homogeneity and hinders the evaporation of the solvent [30]. The presence of air bubbles before the drying process is common due to the mixing steps of the production process; however, it is necessary to remove them, as they lead to the production of films with an irregular surface and a heterogeneous thickness [3].

Drying is the most time-consuming step in the solvent casting production process, which can last from 6 to 48 h [50]; therefore, the choice of solvent is also crucial to control the evaporation rate during the film production. Water, alcohols, organic solvents, or a mixture of solvents such as hydroalcoholic mixtures may be used. Slow drying allows the production of more homogeneous films; however, if the time is excessive until the complete evaporation of the solvent, in some cases, it can lead to microbial proliferation in the solution, and in this case, it is necessary to add preservatives to the formulation [30]. The use of organic solvents is not recommended in the production of films, especially those for application on mucous membranes, due to the risk of residues in the final pharmaceutical form that are harmful to health and the environment [3]. Environmental temperature and humidity conditions also affect film drying; thus, higher temperatures can be used to reduce production time, but the conditions used must take into account the stability and degradation of the formulation components, and it is often impracticable to use high temperatures [30,49].

Despite being a simple process, there are many variables that must be controlled during the production of solvent-casted films. The polymer concentration used is the parameter that most influences the final film thickness, which reflects on the mechanical properties and drug release rate [30]. It is also important to avoid recrystallization of the drug at the end of the process and to guarantee the homogeneity of the film’s thickness so that there is no unequal dose distribution [2].

In solvent casting, it is possible to produce films with several different layers when performing more than one drying step, which allows the incorporation of active pharmaceutical ingredients (APIs) with physical–chemical incompatibilities as well as loading them in different concentrations and in different layers of film [49]. This also allows the production of films with backing layers, drug-loaded reservoirs, and mucoadhesive layers [50].

### 4.2. Electrospinning

Electrospinning is a single-step technique for producing films with a highly porous internal structure formed by nanofibers. The technique consists of pumping a solvent-based polymeric formulation with a controlled flow through a metallic needle under a high-voltage electric current (in the range of 10 to 35 kV) above an oppositely charged collector [49,51]. This technology allows a direct transition from liquid to solid nanofibers and enables the use of various polymers, drugs, functional particles, proteins, and peptides with infinite possibilities of three-dimensional structures, which allows the obtaining of films with different drug release behaviors [52].

The active substance can be added to the film before or after the process; however, in the first case, the drug dissolution rate is better due to the large surface area of the nanofibers [49]. Electrospinning is a simple technology to prepare, and for well-characterized formulations, its results are reproducible. Through this technique, films can be produced in various sizes and shapes and with several layers, both hydrophilic and hydrophobic [49,52]. The process, however, requires that the used fluid has a low viscosity and (in some cases) uses organic solvents, which can generate hazardous waste in addition to taking a longer time and having a higher production cost compared to solvent casting [30,49].

Nadaf et al. [53] recently published a review on nanofiber production processes using electrospinning technology that can be consulted for more details.

### 4.3. Hot-Melt Extrusion

Hot-melt extrusion (HME) film production consists of extruding a mixture of APIs and molten excipients through a matrix in a continuous and reproducible way at temperatures above the glass transition temperature or above the melting temperature. This heating converts the components into a uniform amorphous product, which enhances the dissolution of poorly water-soluble drugs. The final thickness and width of the film depends on the dimension of the die and the geometry and rotation rate of the calender that stretches the extrudate during coil formation [1,3,49]. The HME technique can be used coupled with fused deposition 3D-printing modeling technology for film production [2,54].

The hot-melt extrusion technique, unlike the solvent casting and electrospinning techniques, does not use solvents and therefore does not impact the environment due to the use of volatile organic solvents; it also presents a smaller number of operations than solvent casting and produces films with better content uniformity. Hot-melt extrusion also allows the incorporation of poorly soluble drugs [2,55]. On the other hand, the operating temperatures of the technique are high and limit the APIs and polymers that can be processed using this technology [49]. Other problems encountered in this technique are the possible recrystallization of the drug and swelling of the films at the end of production [2].

### 4.4. Printing Technologies

#### 4.4.1. Inkjet Printing

Inkjet printing is a technology for the deposition of pharmaceutical formulations in droplets on an appropriate support substrate, similar to inkjet printing on paper. The process consists of preparing the impression fluid with the API dissolved or dispersed in a suitable solvent or dispersant that will be deposited in the form of a drop through a nozzle onto a substrate in a programmed manner, followed by a drying process [49,56]. This technology should be used in combination with other film-manufacturing techniques (solvent casting, hot-melt extrusion, or 3D printing) to produce the substrate onto which the API will be deposited; therefore, the API is not incorporated within a substrate, which prevents its crystallization [2].

The most widely used inkjet technique is drop-on-demand. This technology can be performed via the thermal method, in which the drops are ejected by heating the liquid above its boiling point, making it possible to use only water-based fluids; or by the piezoelectric method, in which the voltage applied to a transducer piezoelectric leads to the material’s vibration, making it possible to use both solvent-based and water-based fluids. For the production of films through inkjet printing, the drug must be stable in the printing fluid during production and storage and the support film must be stable during printing, avoiding disintegration and maintaining its mucoadhesive properties [2,56].

Viscosity and surface tension are the most important properties to be considered in the preparation of the printing fluid for the formation of the drop to occur; therefore, it is necessary to add one or more excipients that must be non-toxic and of pharmaceutical grade [56]. Furthermore, during the printing process, nozzle clogging may occur, which leads to dosing errors, as the necessary volume is not ejected correctly [2,56].

Currently, this technology is only applicable when low doses of the active substance are needed [2], but it is advantageous because it allows the precise incorporation of the drug on demand and adaptation to the patient’s needs, depending on the medical prescription. Unlike the solvent casting technique, in inkjet printing it is not necessary to find the exact thickness of the film to control the drug dosage in the final product; instead, it can be controlled by the printing parameters, printing fluid concentration, and number of layers [56].

#### 4.4.2. Three-Dimensional Printing

Three-dimensional-printing technology, which allows three-way printing while adding material layer by layer, resulting in a 3D dosage form [49], has the potential to solve formulation problems in film production. Films might be used for the administration of potent drugs due to the low load capacity of the drug; however, 3D printing allows the deposition of formulation layers on top of each other and is able to incorporate more active substance per unit area of a mucoadhesive film without increasing the size of the pharmaceutical form, which (depending on the application site) can be limited, an advantage of this method. Multilayer deposition can also be used when the ingredients are incompatible or to produce a controlled drug delivery system [2,49]. Film production via 3D printing on a large scale, however, is not yet possible; thus, it is used in the personalized administration of drugs to meet the individual needs of patients or for the concomitant administration of several APIs [57].

Fused deposition modeling is one of the most used techniques in 3D printing. In this technique, the printed object is formed by layers of thermoplastic filaments produced via hot-melt extrusion that are deposited sequentially in a predefined way by a computer. The material is heated inside the printer above its melting point and quickly solidifies after deposition to form a 3D object. This is the most chosen method for manufacturing dosage forms due to its cost-effectiveness, the printing speed, and the large number of pharmaceutical-grade polymers available for printing [2,50].

Three-dimensional printing via semi-solid extrusion involves depositing layers of semi-solid materials using a syringe-like tool. The semi-solids used are mixtures of polymeric substances and appropriate solvent(s) to produce a formulation of suitable viscosity for printing. Unlike fused deposition modeling, semi-solid extrusion does not need heating and can be performed at room temperature using thermosensitive drugs. In addition, objects printed using this technique are wet at the end of printing; therefore, it requires an additional post-printing drying process that can change the final shape, shrink the printed film, or even lead to collapse due to low hardness [2].

## 5. Different Routes of Administration and Applications

### 5.1. Buccal Films

Considering that the oral route of administration is not effective for some drugs, the buccal mucosa stands out as a promising route of transmucosal administration. Avoiding first-pass hepatic metabolism, enzymatic degradation, and hydrolysis by gastric acid; promoting easy administration that allows use by individuals with swallowing difficulties; and allowing immediate withdrawal in the event of severe side effects are the main advantages of this route. In addition, the increased bioavailability of the drug, the reduction in the necessary dose, and the number of daily doses that leads to the occurrence of fewer side effects should be noted. In general, the buccal mucosa has stood out for its high vascularity, absence of enzymatic activity and drug degradation, rapid onset of pharmacological action, and flexibility for use by the patient. It is also worth mentioning that the buccal mucosa is in a region that allows the systemic administration of the drug through the internal jugular vein, preventing the process of hydrolysis in the stomach and hepatic metabolism. Therefore, one of the most used applications for the transbuccal route is to treat acute conditions that require a rapid therapeutic response. In addition, due to its characteristic of immobility, the oral mucosa also presents very promising characteristics for the application of formulations that need to be retained for a longer period, such as controlled-release systems [58,59,60].

However, some important barriers to the delivery of drugs through the buccal mucosa should be highlighted, such as: (1) the presence of elastic fibers in the dermis, which hinders the diffusion of the drug; (2) the continuous secretion of saliva, leading to drug washout and dilution; (3) saliva swallowing or the occurrence of involuntary gagging, which can lead to significant drug loss; (4) the presence of extracellular lipid material derived from the membrane-coating granules; (5) the solubility and potential permeability of the drug, which are important factors to be considered; and (6) the active transport across the mucosa being limited by the action of dendritic cells [58].

There are several commercial forms of buccal administration; these include pills, sprays, sublingual lozenges, chewing gum, films, and oral mucosal solutions [60]. Examples of products currently on the market that exploit the buccal route are buprenorphine hydrochloride soluble buccal film (Belbuca^®^), fentanyl buccal soluble film (Onsolis^®^), fentanyl lozenges (Actiq^®^), fentanyl buccal tablets (Fentora^®^), and a combination of buprenorphine and naloxone (Bunavail^®^), as well as a patch containing lidocaine (Dentipatch^®^) [61].

Buccal films, which stand out for their ease of application for adults and children, are intended for local or systemic therapeutic action. The mechanism of drug retention in this mucosa directly depends on the employed polymers in the formulation, which have different mucoadhesive properties that were previously discussed [60]. Therefore, mucoadhesive buccal films are presented as a very innovative and promising pharmaceutical form that allow the API to be absorbed in a constant and controlled way by the biological barrier due to its adhesion to the buccal mucosa and without the need to be swallowed [62]. They also circumvent important limitations associated with oral therapy such as enzymatic degradation, variable rates of drug absorption along the gastrointestinal tract, and extensive hepatic biotransformation. Furthermore, buccal film formulations can be easily scaled up due to their adaptable nature and feasible production processes such as solvent casting and 3D technology. Finally, it is also worth noting that buccal films are, in general, composed of safe excipients and may be a suitable drug delivery system for the pediatric population [61].

**Figure 2:**
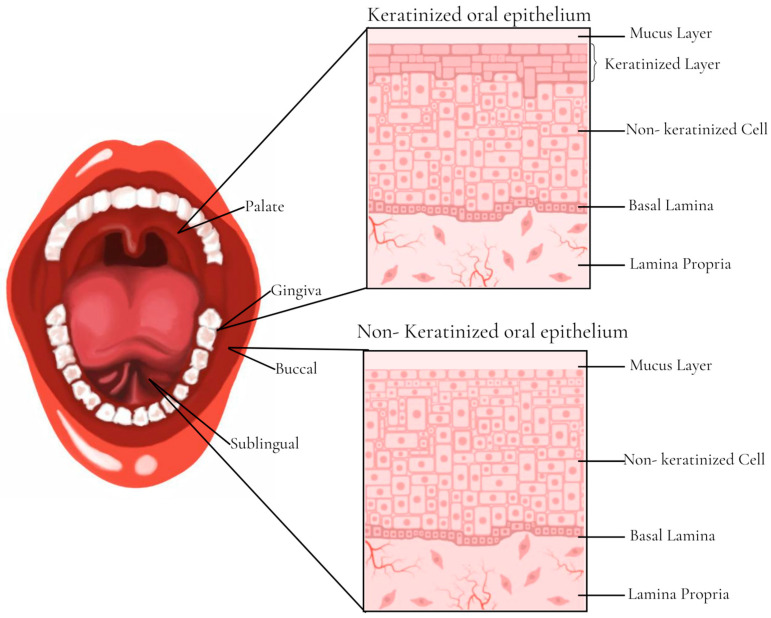
Representation of the buccal structure (**left**) and histological representation (**right**) of the buccal keratinized epithelium mucosa and non-keratinized epithelium mucosa.

The main function of the buccal mucosa (Figure 2) is to protect the underlying tissue from mechanical damage and to act as a primary barrier to food, microbes, and airborne particles. The buccal cavity is lined with a mucous membrane consisting of a layer of stratified squamous epithelium, basement membrane, lamina propria, and submucosa. The epithelium is then composed of five layers: the stratum basal, stratum spinosum, stratum granulosum, and stratum corneum, which is formed by keratinocytes that originate in the stratum basal. In this context, it is in the stratum spinosum that keratinocytes lose their ability to divide and begin the process of terminal differentiation until they reach the stratum granulosum. Once in the granular layer, these cells present granules that extrude lipids, acting as a permeability barrier against hydrophilic materials. Finally, when they reach the stratum corneum, these keratinocytes lose their nuclei and increase keratin production or else remain nucleated and become non-keratinized before being eliminated via desquamation from the oral cavity [63].

The composition of the region of the hard palate, dorsum of the tongue, and gingiva is based on the keratinized stratified squamous epithelium (masticatory mucosa), while the region of the labial mucosa, buccal mucosa (cheeks), soft palate, and floor of the mouth is composed of the non-keratinized stratified squamous epithelium, also called the lining mucosa. Whereas the masticatory mucosa requires a keratinized epithelium strongly attached to the underlying tissues via collagen-containing connective tissue, the lining mucosa (composed of the buccal and sublingual region) consists of a non-keratinized epithelium that is supported by a more flexible and elastic connective tissue. In addition to keratinocytes, which represent 95% of cells in the oral epithelium, there are also dendritic cells that are important in the immune response process and Merkel cells with sensory functions. In addition, the mucins (glycosylated proteins) present on the surface of the epithelium and the inorganic salts secreted by the sublingual salivary glands are also important, as they promote the gelation of the external layer for protection and lubrication of the mucosa, followed by an additional coating of saliva [63,64].

Epithelial cells are interconnected by tight junctions (occluding junction), gap junctions, and anchoring junctions (desmosomes and adherent junctions). It is important to point out that the interactions between these cells are essential for the physiological processes of the oral mucosa, but they can be quickly rearranged under different physiological and pathological conditions. Tight junctions are located in the superficial layers of the stratified oral epithelium and are responsible for keeping the adjacent epithelial cells together, with the main function of preventing the passage of dissolved molecules, microorganisms, and toxins through the epithelial layer. Adhering junctions and desmosomes are located below tight junctions and can maintain cell–cell adhesion through binding to the cytoskeletal filaments that provide maintenance of tissue integrity. Proteins that mediate direct interactions between adjacent cells are present in the adhesion nucleus of junctions, while the proteins that are located in the cytoplasmic region are coupled to the cytoskeleton by means of several adapter proteins. Gap junctions are clusters of intercellular channels that promote a direct connection between the cytoplasm of two neighboring cells and play important roles in maintaining the development and normal function of the oral tissue [64,65,66,67,68].

In the epithelium basolateral layer is the fibrous connective tissue that makes up the lamina propria, where oral fibroblasts produce elastin and collagen fibers to form the extracellular matrix. In addition, blood vessels, glands, and nerves are also located in this region. Below the lamina propria is the submucosa that is present in some regions of the oral cavity to connect the oral mucosa to the underlying muscles through loose connective tissue [63].

#### 5.1.1. Current Applications of Buccal Films

According to the PubMed database, in the last 5 years there were about 37 manuscripts involving the study of buccal films for transmucosal application. When compared to conventional oral solid dosage forms, studies highlight the importance of these formulations for easy application in the elderly, children, and individuals who have difficulty swallowing. In general, the described buccal films stand out due to the significant increase in the bioavailability of the used drugs, which is due to factors such as avoiding first-pass hepatic metabolism, rapid absorption of the molecule by the buccal mucosa, and an increased solubility of the molecule and rate of dissolution, and they even improve the patient’s adherence to pharmacotherapy.

Based on a brief analysis of these studies, the most cited polymers were HPMC, polyvinyl alcohol (PVA), polyvinyl pyrrolidone (PVP), and sodium alginate (SA), while in relation to production methods, solvent casting was the most used technique. Finally, the health conditions mentioned were: (a) hypertension [69,70,71,72,73,74,75], (b) psychological disorders [76,77,78,79,80], (c) migraine [61,81,82,83,84,85], (d) allergic conditions [85,86,87,88,89,90], (e) vomiting and nausea [91,92], (f) diabetes [60,93,94], (g) menopausal symptoms [95,96], (h) erectile dysfunction [97], (i) tuberculosis [98], (j) Alzheimer’s [99], (k) smoking [59], (l) heart failure [100], (m) epilepsy [82], (n) HIV [101], and (o) sedation [102]. Below, we describe examples of recently published studies that were successful in the development of buccal films.

##### Fast-Dissolving Film of Levocetirizine Dihydrochloride

In a study by Al-Kubati et al. [90], the authors aimed to develop solid, dispersed, fast-dissolving films (FDFs) of levocetirizine dihydrochloride by using gelatine as a dispersing agent for LCD and hydroxypropyl methylcellulose as a film-forming agent, with the purpose of treating the symptoms of allergic conditions. Oral fast-dissolving films are formulations composed of hydrophilic polymers that quickly dissolve or disperse when in contact with the oral mucosa. In this context, the film is capable of being instantly hydrated by saliva, which consequently adheres to the mucosa and releases the drug for absorption by the highly vascularized region of the oral mucosa.

Seven FDF compositions were prepared via the solvent casting method containing LCD, HPMC as a film-forming polymer, propylene glycol as a plasticizer, three superdisintegrants (sodium starch glycolate, croscarmellose sodium, and crospovidone), citric acid as a production stimulator of saliva, sucrose as a sweetener, and vanilla as a flavoring agent. The drug was characterized via Fourier transform infrared spectroscopy (FTIR) and Raman spectroscopy; after producing the films, a series of assays were employed to characterize them, such as those to test its thickness, weight variation, folding endurance, surface pH, drug content uniformity, in vitro disintegration time, moisture absorption and loss, and in vitro dissolution, ending with in vivo palatability and disintegration studies as well as those regarding the formulation stability. This study covered all the essential stages of film characterization, including the final stage of clinical study on individuals.

As a result, the authors emphasized that the addition of the drug in the form of a solid suspension in gelatin made it possible to obtain films with desirable characteristics such as flexibility, uniformity, and a smooth surface. Furthermore, it was observed that the films’ weight and thickness were uniform and the bending strength values were greater than 300, demonstrating good flexibility and an ability to resist breaking. Regarding the in vivo studies, there was a rapid disintegration of the films, which can be explained by the movement of pressure of the tongue against the palate, which promoted the film fixation, as well as by the addition of citric acid, which led to an increase in saliva secretion. Finally, the stability tests were also satisfactory in that during the storage period of three months at 45 °C and humidity of 75 ± 5% RH, no changes in the important characteristics of the films were observed.

Therefore, an FDF containing levocetirizine dihydrochloride was successfully obtained and characterized at a 1:1 weight ratio. The film demonstrated rapid drug disintegration and release (30 s and 1 min, respectively) in addition to remaining stable during the storage period. Still, the taste was efficiently masked through the method of solid dispersion of the drug and the addition of flavoring. Thus, a palatable and fast-acting FDF of levocetirizine dihydrochloride was obtained to alleviate the allergic symptoms of allergic rhinitis and inflammation of the upper respiratory tract.

##### Mucoadhesive Film for Rizatriptan

In a study by Nair et al. [61], the objective was to propose a mucoadhesive buccal film containing rizatriptan as a promising alternative strategy for the conventional treatment of migraine. Knowing that the clinical symptoms reported during migraine are intense and complex and that this is the third leading cause of disability in men and women under 50 years of age, the objective of this study was to provide a therapy with a rapid onset of action and of long duration that could be obtained through buccal mucosa rizatriptan administration.

For the development of the buccal mucoadhesive film through the conventional method of solvent casting, Proloc^®^ 15, HPMC, and Eudragit^®^ RS 100 were used as film-forming polymers, propylene glycol and polyethylene glycol 200 as plasticizers, and Tween^®^ 80 as the agent solubilizer. The formulation was evaluated for thickness; pH; drug content; folding endurance; mucoadhesive strength; percent hydration; possible drug–excipient interaction via Fourier transform infrared spectroscopy, differential scanning calorimetry; and scanning electron microscopy; and drug release. Finally, ex vivo permeation and in vivo studies in male rabbits were also carried out. This work obtained a complete characterization of the film and can thus evolve to a more advanced stage of product development, which is the in vivo model study.

In the analysis of the characterization’s obtained results, the films under development showed a thin and comfortable thickness for application on patients, a neutral pH that avoided any sensitivity or allergic reaction to the buccal mucosa, a high resistance value, and an adequate mucoadhesive strength contributed by Proloc^®^ 15 and HPMC. This might be explained by the presence of carbomer in the Proloc^®^ polymer, which allowed the retention of the film in the buccal mucosa for a long period. Furthermore, the authors concluded that the polymer swelling allowed the polymer chains to unwind, promoting hydrogen bonding or electrostatic interactions with the mucin of the oral mucosa. Additionally, the variation in the polymeric composition did not influence the content of this drug, and the drug release profile proved to be biphasic; that is, a higher drug release rate was obtained in the first two hours, so the authors pointed out that this type of profile will ensure good availability of the drug to be absorbed by the mucosal surface. An almost complete release of the drug in the final film formulation was obtained in approximately 6 h. Finally, the in vivo model showed that the buccal therapy allowed an increase in the level of rizatriptan, indicating that there was a good permeability of the drug via the mucosa.

Therefore, it was concluded that the obtained films exhibited excellent physical, mechanical, and pharmaceutical characteristics, showing a complete release of the drug in vitro and a greater permeation of the drug in the ex vivo model, and the formulation components and rizatriptan were compatible. In vivo studies showed a significant increase in the plasma levels of the drug, which indicated an improvement in the extent of absorption, increasing its clinical efficacy, so the film can be considered a potential and effective approach for migraine treatment.

##### Three-Dimensionally Printed Bilayer Mucoadhesive Buccal Film of Estradiol

Abdella et al. [95] proposed the development of an innovative 3D-printing technology to design a bi-layered estradiol film with different infill patterns with the aim of improving bioavailability and facilitating personalized treatment for menopausal symptoms. Among buccal dosage forms, buccal films composed of ultrathin strips constituted by one or several layers of suitable material are preferred due to their higher flexibility, more accurate dosing, and reduction in side effects. In addition, they guarantee the unidirectional flow of the drug through the application of a support layer, which increases the drug permeation and bioavailability, avoiding the washing process by saliva.

According to the authors, there was a previous study by the group regarding a buccal film containing estradiol produced via the solvent casting method; however, the formulation showed a non-uniformity of the drug due to the viscosity and spreadability issues of the formulation. Additionally, there was also the limitation of the inability of this method to prepare films that meet the individual clinical needs of each patient, which can be obtained via the 3D-printed method that allows changing the format, size, dose, color, and release kinetics of the film. It is also worth mentioning that the 3D production method allowed adding specific structural properties with the introduction of drug-loaded reservoirs, support layers, and mucoadhesive layers, which is a challenge to be considered in solvent casting. In this way, the study involved the use of the pressure-assisted microsyringe (PAM) technique of a double nozzle to develop a buccal film of estradiol with two layers; this method of 3D printing uses material extrusion that creates objects through the sequential deposition of gel or paste layers.

The backing layer was prepared from the formulation based on hydroxyethyl cellulose (HEC) with some modifications. Then, the drug was incorporated into the apical layer so that a nanoformulation was obtained with a mixture of estradiol, polyethylene glycol, Transcutol^®^ P, Tween^®^ 80, and water to increase the solubility and permeability characteristics of the drug. Different polymers were used alone or in combination for 3D printing: PVA, HPMC E50, Carbopol^®^, and HPC-H. Sorbitol was then used as a sweetening agent, filler, and plasticizer. For the characterization of the obtained films, several evaluation tests were carried out: physical appearance, mechanical and mucoadhesion properties, content uniformity, FTIR, differential scanning calorimetry (DSC), X-ray powder diffraction (XRD), scanning electron microscopy (SEM), in vitro dissolution, determination of the mechanism of drug release, and in vivo performance prediction. Despite presenting several results of film characterization, the study has not yet advanced to the in vitro drug permeation analysis stage; consequently, there has not yet been an analysis of the in vivo release profile. Therefore, this work is still in the actual study stage of the formulation development.

The obtained films showed good mechanical properties, no differences in the mechanical strength between the different film formats and with the inclusion of the drug, and good mucoadhesion; the authors attributed this characteristic to the PVA presence. The analysis of the drug content led to the conclusion that by increasing the thickness, size, or pressure, a higher drug dose could be obtained. In addition, spectroscopy, calorimetry, and X-ray tests confirmed that the drug was able to be incorporated into the formulation with no interaction between the components and a good molecular dispersion.

According to the authors, the drug release rate findings for the formulation were determined through a complex process that depended on several factors, such as filling pattern and density, type of polymers and excipients used, and the drug properties. Interestingly, films with rectangular and honeycomb fill patterns released the drug faster than films with simple fill patterns, highlighting the impact of fill patterns on the release kinetics and demonstrating the importance of 3D printing in this regard. Therefore, this study reinforced the need to understand the release profile of each pharmaceutical form and adapt the formulation to the reality of each patient.

### 5.2. Nasal Films

Due to the high vascularity of the nasal mucosa and the permeability of its epithelium, the nasal route is a promising non-invasive route for the administration of drugs with local or systemic action. This route has advantages over the oral or intravenous ones because it allows self-administration for greater patient comfort and it has a shorter time for the onset of the pharmacological effect and greater bioavailability (since it is not affected by the first-pass hepatic metabolism) [103,104,105]. Nasal administration of drugs and other components that are difficult to administer through systemic circulation is possible; these include low-molecular-mass compounds with high polarity, peptides, and polysaccharides; or high-molecular-weight proteins that are components of vaccines, including DNA plasmids [103]. The nasal route also allows the direct absorption of molecules by the brain through the olfactory region or the trigeminal nerves that innervate the nasal cavity, favoring the administration of drugs to the central nervous system (CNS) with greater bioavailability without interference by the blood–brain barrier [104].

The main factors that influence the absorption of drugs through the nose are: (1) the physicochemical characteristics of the drug molecule itself, such as the partition coefficient, pKa, molecular mass, perfusion rate, acidity of the solution, and concentration of the drug; (2) the action of the mucociliary system within the nose (which depends on the age and sex of the patient, whether they are asleep or awake, and whether they are resting or exercising), air pollution, diseases (such as rhinitis, bronchitis, and asthma), and the use of medications; and (3) the presence of any factors that increase nasal absorption [103].

Mucociliary clearance is a defense mechanism of the nasal cavity and may influence the permanence time of the formulation. Through this mechanism, the mucus and substances adhered to the mucosa are cleaned and drained to the nasopharynx, where they will be discharged into the gastrointestinal tract; therefore, substances administered through the nose are eliminated from the nasal cavity in 21 min [105], which suggests that the administration of mucoadhesive films that remain for a longer period in the nasal cavity is promising.

**Figure 3:**
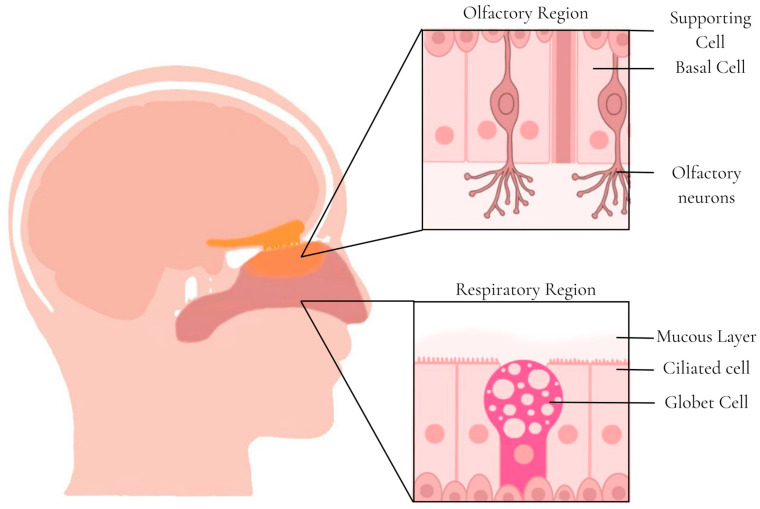
Representation of the nasal structure (**left**) and histological representation (**right**) of the olfactory region mucosa and respiratory region mucosa.

The nose is composed of two symmetrical cavities divided by the nasal septum, with the nostrils at the anterior entry and the choanae at the posterior end [105] (Figure 3). The entire internal cavity is lined with a layer of mucosa with a total area of 150 cm^2^ that can be divided into three main regions: vestibular, respiratory, and olfactory [106]. The vestibular region is the most anterior (just beyond the openings of the nostrils) and has a small surface area that contains numerous nasal hairs that contribute to the filtration of large inhaled particles. The tissue in the buccal region is scaly with few or no hair cells, and the reduced surface area accounts for minimal drug absorption in the region. The respiratory region has the largest area (approximately 130 cm^2^) and is the most vascularized, thus making it the region with the greatest systemic absorption of drugs. This region has goblet, basal, and columnar ciliated and non-ciliated cells. Goblet cells are responsible for secreting mucin into the mucus layer, and columnar cells help increase the surface area. The maxillary branch of the trigeminal nerve innervates the respiratory region, a possible target for the transport of drugs to the CNS [105,106].

The olfactory region is in the upper part of the nasal cavities and has a small area of 10 cm^2^ with four types of cells: basal, supporting, ophthalmic branch of the trigeminal, and olfactory neurons. Olfactory neurons are unmyelinated bipolar cells that project cilia into the mucous layer to transduce olfactory signals [106]. These neurons also project axons into the olfactory bulb, allowing the blood–brain barrier (BBB) to be circumvented for direct drug delivery to the CNS (nose-to-brain delivery). Innervation by the trigeminal nerve in this region also enables transport to the CNS [105].

#### 5.2.1. Current Applications of Nasal Films

Through searches in PubMed, only five research articles were found with films applied to the nasal mucosa in the last 5 years. The studies highlighted the importance of applying films to the nasal mucosa to increase the drug’s residence time at the site, to avoid first-pass metabolism by the liver, and to administer drugs to the CNS (nose-to-brain). Of the five manuscripts found, one of them indicated a film application for the treatment of dry nasal syndrome [7], therefore representing a local action film that did not fit the objectives of this review, while another one [107] described the development of films using low solubility anti-inflammatory drugs as a model drug without indicating the applicability of the films; it therefore did not fit in this review either.

The polymers used to manufacture the films were PVA and HPMC, the latter being the most cited. In all studies, the production method used was solvent casting and the intended administration was through the blood–brain barrier to reach the CNS; two of them were used for Alzheimer’s disease treatment [108,109] and the other was used for treatment of anosmia in patients after COVID-19 infection [110]. Below, we will explore an example of recently published studies that were successful in the development of nasal films.

##### Mucoadhesive Nasal Film for Nose-to-Brain Delivery of Donezepil

Papakyriakopoulou et al. [108] developed HPMC films containing donepezil hydrochloride, a reversible acetylcholinesterase inhibitor (AChEI), for nose-to-brain administration for treatment of Alzheimer’s disease. PEG 400 was used as a plasticizer, and methyl -β- cyclodextrin (Me-β-CD) was used as an absorption promoter. The films were produced via solvent casting using cylindrical blisters as molds.

The films were characterized according to donepezil hydrochloride content, film thickness, folding endurance, percent moisture loss, and a swelling test. Stability experiments, donepezil hydrochloride in vitro drug release experiments, and mucoadhesive ability and permeation experiments were also performed. The mucoadhesive ability and permeation experiments were performed using rabbit nasal mucosa as the model barrier. The optimal nasal film for donepezil hydrochloride delivery was determined via Response Surface Methodology.

Most of the produced films remained stable for a period of 6 months, and the release studies showed that the absorption promoter interfered with the drug release; however, in the permeation studies, the absorption promoter increased the percentage of the drug that permeated through the rabbit nasal mucosa. Regarding the mucoadhesion experiments, all films remained stationary on the placement site for 2 h.

### 5.3. Ocular Films

The ocular mucosa presents a challenging environment for the administration of drugs, being necessary to overcome many barriers and mechanisms that limit the penetration of the eye. These barriers are very important for ocular protection; however, they make the drugs’ bioavailability in the ocular tissue low [8,111]. Low bioavailability is caused by: (1) the compact cell layers of the corneal epithelium, which hinder the passage of hydrophilic and ionized drugs; (2) the corneal stroma, which constitutes a hydrophilic space between the epithelium and the endothelium and hinders the passage of lipophilic compounds, and (3) the drainage of lacrimal fluid into the nasolacrimal duct induced by blinking [58].

Due to the ease of administration, the most used pharmaceutical form for application in the eye is the eye drop; however, less than 5% of the drug reaches the deeper ocular tissues, making it very difficult to reach the therapeutic concentration in the posterior segment of the eye. For drug delivery in the posterior segment, the application can be performed with periocular injections, intravitreal injections, and intraocular inserts. Intravitreal injection is the most used treatment for diseases of the retina and choroid; however, the application of ocular injections may cause endophthalmitis, hemorrhage, retinal detachment, and low patient tolerance. Systemic administration, on the other hand, is not used in the treatment of ocular diseases, as ocular penetration is very limited by the retinal blood barriers and the dose required for systemic administration would have to be very high, leading to the risk of systemic toxicity [8].

Ocular drug delivery solids have potential benefits over liquids, such as better storage stability and greater ocular bioavailability, as they are able to form a transparent film with a longer residence time in the ocular mucosa [111]. The films must, however, be comfortable and transparent to not to interfere with the patient’s vision.

**Figure 4:**
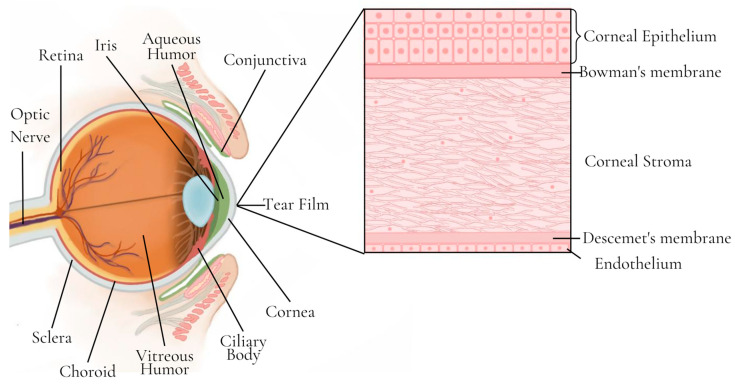
Representation of the ocular structure (**left**) and histological representation of the ocular mucosa (**right**).

The outer layer of the eye is made up of the sclera at the back, the cornea at the front, and the conjunctiva, which is a membrane that covers part of the sclera and extends into the eyelids (Figure 4). The tear film, produced by the lacrimal gland, covers the surface of the cornea and conjunctiva and is composed of the mucous layer (which interacts with the corneal epithelial cells), the aqueous layer, and the lipid layer. The middle layer of the eyeball is formed by the iris, the ciliary body, and the choroid, while the inner layer is the retina. Between the retina and the sclera is the choroid, the vascular layer of the eye that is responsible for supplying oxygen and nutrients [58,112].

#### Current Applications of Ocular Films

Nine manuscripts related to ocular transmucosal films were found in the PubMed search over the past 5 years. The studies highlighted the importance of developing sustained-release formulations for the ocular route due to the low bioavailability of drugs resulting from the topical application of liquid formulations in this region.

A wide variety of polymers were used for the manufacture of these films, such as: hydroxypropyl cellulose (HPC), polycaprolactone, hyaluronic acid, hydroxypropyl methylcellulose, poloxamer 407, polyurethane, polyvinyl alcohol, polyvinyl acetate, polyethylene oxide, and gelatin. The most cited polymer in these studies was polycaprolactone. The production methods used were hot-melt extrusion, solvent casting, and electrospinning, the latter being the most used for the production of ocular films in the most recent research. The health conditions indicated for treatment were glaucoma [113,114,115,116], inflammation of posterior section of the ocular tissues [117,118,119,120], and age-related macular degeneration [121].

A successful example of ocular film was created by Andreadis et al. [115] in a study that aimed to address the shortcomings of ophthalmic drug solutions by developing electrospun nanofiber films composed of PVA and Poloxamer 407 as film-forming polymers and timolol maleate for the management of glaucoma. Nanofibrous films are capable of readily adapting to the cornea surface and dissolving, making them promising for ocular application due to the comfort of the patient during their time in the mucosa.

In this study, the films produced were characterized morphologically via scanning electron microscopy and physicochemically via their infrared spectra, X-ray diffraction, and thermal behavior. Experiments regarding the rheological characterization of the hydrated films and the Hen’s Egg Test on Chorioallantoic Membrane (HET-CAM) were performed. In the ex vivo studies, they assessed the corneal permeability followed by corneal tissue characterization, drug accumulation in the corneal tissue, and histological studies. In the in vivo studies, they assessed the ocular tolerance by using the Draize eye test and intraocular pressure measurements.

The developed formulation showed practically no irritation, hyperemia, hemorrhage, or coagulation in the HET-CAM test and was characterized by the Draize test as practically non-irritating. The tested films presented higher drug permeation through the cornea than the drug solution. All formulations induced an IOP-lowering effect for a longer time than the drug solution and resulted in an earlier onset of action as well. This is a desirable effect that could lead to a reduction in the numbers of doses and the dose frequency in glaucoma treatment, thereby increasing patient compliance, especially among elderly patients.

According to the authors, these in situ gelling ocular films could serve as an effective alternative to conventional eye drops in the treatment of glaucoma. The formulations proved to be safe for ophthalmic application and have the potential for a greater permeability and prolonged action of the drug, contributing to patient compliance.

### 5.4. Vaginal Films

The vaginal route has a large surface area with a high blood supply and avoids first-pass hepatic metabolism, making it a promising route for administering drugs for systemic effect [112,122] and for local administration of drugs that prevent sexual viral transmission. Vaginal administration of drugs, however, is influenced by the patient’s physiological characteristics, such as the vaginal pH, microflora, and enzymatic activity [122]. Vaginal pH ranges from 3.8 to 4.5; however, women after menopause or before menstruation have a vaginal pH around 5.0, and the presence of semen can lead to a vaginal pH above 6.0. Depending on the drug and its pKa, these conditions affect its release and permeation [122,123].

Most conventional vaginal formulations (creams and gels) leak prematurely [122], decreasing the drug’s residence time at the site of action or the percentage of drug that reaches the systemic circulation. Vaginal films prevent the formulation from leaking, in addition to being small, discreet, easy to store and administer without the need for an applicator, more flexible and comfortable than pills, and allowing for accurate dose administration [30,123].

**Figure 5:**
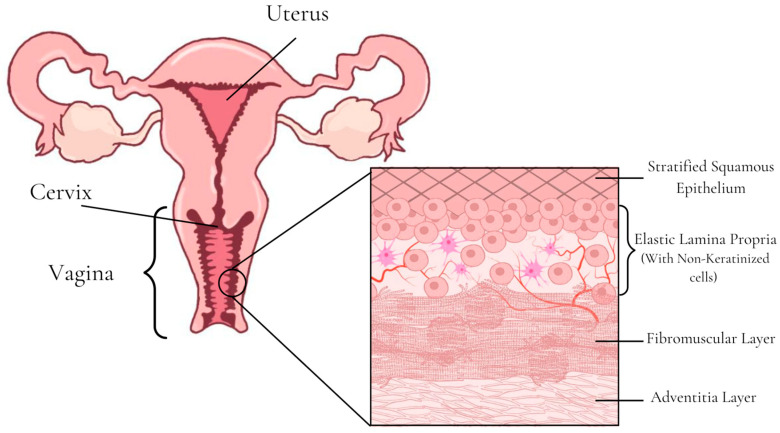
Representation of the vaginal structure (**left**) and histological representation of the vaginal mucosa (**right**).

The human vagina has a tubular-shaped fibromuscular structure that connects the uterus to the external environment, and its wall is lined with mucus or cervical–vaginal fluids [112]. Histologically, the mucosa (Figure 5) has four different layers: the stratified squamous epithelium, the non-keratinized elastic lamina propria, the fibromuscular layer with two layers of smooth muscle, and the outer adventitial layer. Changes in estrogen levels can influence the vaginal epithelium’s thickness, which is normally 200–300 μm thick [58].

#### Current Applications of Vaginal Films

In the search carried out in PubMed referring to the last 5 years of publications, several articles were found about films for vaginal administration. The most cited applications were prevention of HIV transmission [124,125,126,127,128], candidiasis [129,130,131], vaginal microbial infections [132,133,134], cervical cancer [135], and genital herpes [136]. However, these studies only dealt with drugs for local action or did not indicate the site of action and therefore were not considered in the present review. Only the one developed by Li et al. [137], which regarded a multipurpose prevention technology film with a systemic contraceptive action concomitant with local action to prevent HIV transmission, will be discussed later.

In the aforementioned study, the authors highlighted the importance of multipurpose prevention technology for preventing the spread of sexually transmitted infections (STIs), including HIV, and unintended pregnancies using films because of their low price, discreet appearance, ease of transport, and ease of self-administration by women. This was a proof-of-concept study to describe the development of an innovative bioadhesive film platform for the combined delivery of an antiretroviral drug and a progestin. The films were produced via the solvent casting method using PVA, HPMC, PEG 8000, and a modified chitosan with thiol groups (thiomers) as film-forming polymers, glycerin and propylene glycol as plasticizers, and sodium starch glycolate as the disintegrant. The drugs used were dapivirine and levonorgestrel.

The authors performed in vitro dissolution tests, ex vivo tissue mucoadhesion tests, and in vivo evaluations in macaques. The results demonstrated that the developed film was able to remain in the vaginal mucosa of the macaques for 7 days and that the drug release was sustained during this period in both the in vivo and in vitro assays. The results also showed a sufficient dapivirine concentration in the vaginal fluid and vaginal and cervical tissues (even with co-delivery of levornorgestrel), and the levornorgestrel concentration for the combination film as compared to the single-entity one still was higher in the plasma and lower in the vaginal fluid.

Table 1 shows some other examples of current applications of oral, nasal, and ocular films with different production methods.

## 6. Final Considerations

In making a general overview of the different points addressed in this review, some aspects concerning the most relevant characteristics deserve to be considered regarding the use of films as transmucosal drug delivery systems.

The most used polymer for film production in the recently published studies was HPMC. HPMC is a polymer derived from cellulose that is biocompatible and water-soluble, is cleaved by enzymes, and is neutral; therefore, its solubility is not influenced by pH, making it a stable polymer in a wide range of pH (3 to 11). Both in powder form and in solution, HPMC is odorless and tasteless, which makes it an excellent polymer for use both in the pharmaceutical industry and in the food and cosmetic industries. There are several HPMC suppliers that offer the polymer with different molecular weights, viscosities, particle sizes, and chemical structures, enabling a wide variety of applications. In addition to the aforementioned advantages, HPMC is an excellent candidate for film production due to its: (1) mucoadhesion properties, which occur through absorption and diffusion mechanisms; (2) ability to swell and form a gel layer, controlling the rate of drug release; (3) non-ionic nature, which ensures a minimal risk of drug interactions and generally provides reproducible drug release profiles; and (4) thermoplastic behavior, which enables the use of the polymer for film production through hot-melt extrusion and 3D printing [8,138].

Except for the ocular route, the most used film production method was solvent casting. The solvent casting method is not the ideal production method for all formulations due to the many variables that must be controlled during the process and due to the risk of API recrystallization; however, it is a simple, well-known, low-cost method that allows the use of various APIs and polymers, as it does not depend on the use of high temperatures, and was therefore the most used method [2,30]. In the ocular pathway, on the other hand, the most used technique was electrospinning. The nanofiber films produced by electrospinning have a soft and flexible nature, porous structure, and good mechanical strength, increasing patient comfort and consequently increasing their adherence to the treatment. In addition, nanofibers have a high surface-to-volume ratio, allowing the release of a greater amount of APIs per area so that it is possible to cross the barriers of the ocular mucosa [139].

The vaginal and nasal routes were the ones that presented the lowest number of studies of films for transmucosal administration. The vaginal route has advantages for the application of films with local and systemic action, as it has a large surface area and a rich blood supply, it avoids the hepatic first-pass effect, it has a relatively high permeability to many drugs, and it allows self-insertion; however, during the development of vaginal films, some disadvantages must be considered, such as cultural background, personal hygiene, gender specificity, local irritation, pH variation, and influence on sexual intercourse. Vaginal films are designed to rapidly disperse or dissolve to form a smooth, viscous mucoadhesive gel with a good appearance and must be soft, flexible, and without sharp edges to be easily inserted into the vagina to increase patient acceptability and compliance. As vaginal films do not cause discharges like other vaginal formulations, they are more accepted by women [140]; however, they are more frequently used for local action such as HIV prevention, since systemic action films are more easily applied to the buccal mucosa.

For the same reason, there were no recent studies with systemic action films for nasal application, and the studies of nose-to-brain administration films were limited. Currently, studies of nose-to-brain administration are performed with liquid or powder formulations that can be applied with a spray. Recently, a nasal applicator for nose-to-brain administration films was developed. In this study, the applicator was produced via fused deposition modeling 3D printing and tested in a nasal cast with an in situ film-forming gel formulation [141]. With the development of nasal applicators, it is possible that new studies of nasal application films will be carried out, since they are a promising pharmaceutical form for administering drugs through the nose-to-brain route.

Therefore, films are promising pharmaceutical forms for transmucosal drug delivery. The oral administration films are well-studied pharmaceutical forms; however, there are still opportunities for studies on films administered in other mucosa, mainly for application in the ocular and nasal routes. The development of nanofiber films allows a greater release of drugs per area; therefore, they are extensively researched for ocular application. However, no studies were found on nasal application, which is a promising region for the application of nanofibers. The application of films in the nasal cavity is difficult due to the anatomy of the nostrils; however, the development of nasal film applicators allows for easier administration, supporting new research in this area.

Finally, 3D-printing technologies are still little explored for film management due to the difficulty of large-scale production. These technologies are promising for the development of films for personalized therapies and for drugs with a narrow therapeutic window, as they allow greater control of the administered dose, therefore making them excellent techniques in the development of future research.

## Figures and Tables

**Table 1:** Examples of current application of film drug delivery systems considering the aspects of production method, composition, and application site.

Drug Delivery System	Production Method	Polymer	Administration Site	Results
Nanoparticulate, oral, fast-dissolving film dosage form of nitrendipine [58]	Solvent casting	HPMC E6 and PVA	Buccal	Increase solubility and dissolution of the drug
Polymeric and electrospun patches for drug delivery [66]	Solvent casting	PVA, sodium alginate, and sodium carboxymethyl cellulose	Buccal	Excellent mucoadhesive strength; buccal patch had optimum pH for buccal compatibility and prolonged release up to 5 h
Hydrobromide immediate-release buccal films using a central composite rotatable design [73]	Solvent casting	HPMC E5	Buccal	Immediate-release drug delivery, rapidly improved migraine conditions, and enhanced patient compliance
Insulin fast-dissolving film [98]	Solvent casting	HPMC and PVA	Nasal	Significant elevation of the olfactory detection scores and olfactory discrimination values in the intervention group after intranasal administration
Timolol-loaded composite [103]	Solvent casting	Hyaluronic acid and HPMC	Ocular	Prolonged drug release profiles
Buccal films of propranolol hydrochloride [60]	3D Printing	PVP and PVA	Buccal	Good mechanical and mucoadhesive pro-perties and prolonged drug release
Solid, dispersed, multilayered core-sheath, raloxifene-loaded, nanofibrous [84]	Electrospinning	PVA, HPMC, and chitosan	Buccal	Ongoing studies with expected therapeutic potential for use in preventing and treating osteoporosis and decreasing the risk of developing invasive breast cancer in postmenopausal women.
Nanofibrous fluocinolone acetonide [109]	Electrospinning	Poly(caprolactone)	Ocular	Higher drug permeation, higher systemic availability of drug, and higher retention of the drug carrier at the absorption site
Midazolam (2-dimensionally printed) [90]	Inkjet printing	Kollidon^®^ 12 PF (polyvinyl pyrrolidone)	Buccal	Increased absorption and residence time of midazolam in the oral cavity

## Data Availability

Not applicable.
